# Stability of small pegs for cementless implant fixation

**DOI:** 10.1002/jor.23572

**Published:** 2017-05-23

**Authors:** Diogo M. Geraldes, Ulrich Hansen, Jonathan Jeffers, Andrew A. Amis

**Affiliations:** ^1^ Biomechanics Group, Department of Mechanical Engineering Imperial College London Exhibition Road SW7 2AZ London United Kingdom; ^2^ Musculoskeletal Surgery Group, Department of Surgery and Cancer Imperial College London School of Medicine W6 8RF London United Kingdom

**Keywords:** cementless implant fixation, interference fit, press‐fit, shoulder, glenoid

## Abstract

Most glenoid implants rely on large centrally located fixation features to avoid perforation of the glenoid vault in its peripheral regions. Upon revision of such components there may not be enough bone left for the reinsertion of an anatomical prosthesis. Multiple press‐fit small pegs would allow for less bone resection and strong anchoring in the stiffer and denser peripheral subchondral bone. This study assessed the fixation characteristics, measured as the push‐in (*P*_in_) and pull‐out (*P*_out_) forces, and spring‐back, measured as the elastic displacement immediately after insertion, for five different small press‐fitted peg configurations manufactured out of UHMWPE cylinders (5 mm diameter and length). A total of 16 specimens for each configuration were tested in two types of solid bone substitute: Hard (40 PCF, 0.64 g/cm^3^, worst‐case scenario of *P*_in_) and soft (15 PCF, 0.24 g/cm^3^, worst‐case scenario of spring‐back and *P*_out_). Two different diametric interference‐fits were studied. Geometries with lower stiffness fins (large length to width aspect ratio) were the best performing designs in terms of primary fixation stability. They required the lowest force to fully seat, meaning they are less damaging to the bone during implantation, while providing the highest *P*_out_/*P*_in_ ratio, indicating that when implanted they provide the strongest anchoring for the glenoid component. It is highlighted that drilling of chamfered holes could minimize spring‐back displacements. These findings are relevant for the design of implants press‐fitted pegs because primary fixation has been shown to be an important factor in achieving osseointegration and longevity of secondary fixation. © 2017 Orthopaedic Research Society. Published by Wiley Periodicals, Inc. J Orthop Res 35:2765–2772, 2017.

Glenoid component loosening is the leading cause of shoulder replacement failure, responsible for up to 63% of revision surgeries.^1^ Implant fixation is commonly achieved through the use of polymethylmethacrylate (PMMA) bone cement. Exothermic bone necrosis can occur during setting of the PMMA^2^ and high incidence of radiolucent lines has been observed.^3^ Furthermore, cyclic loading during performance of daily activities causes failure of the implant‐cement interface.^4^, ^5^, ^6^ Metal‐backed implants have been suggested as an alternative to cemented fixation because of the ability to achieve a porous bone ingrowth surface,^7^ but dissociation of the ultra‐high molecular weight polyethylene (UHMWPE) liner from the metal tray^8^ or wear of both parts have resulted in unacceptably high failure rates.^3^, ^9^

Implants relying on directly press‐fitting UHMWPE onto bone show acceptable short term results^10^ and are used despite the lack of long term survival data.^11^ Press‐fitted acetabular implants coated with a layer of titanium particles promote osseointegration,^12^ have excellent long‐term survival in the hip^13^ and could be a promising technological advance in shoulder arthroplasty.^14^

Translation of the humeral head on the glenoid implant surface results in edge loading, forcing the implant to tilt and pull out its fixation features.^15^, ^16^ This mechanism has been identified as the main cause of glenoid fixation failure and is known as rocking horse movement.^17^, ^18^ Poor primary fixation immediately after implantation^19^ or improper implant seating due to elastic spring‐back displacement of press‐fitted pegs^14^ results in large micromotion at the bone‐implant interface under such loading conditions. This leads to formation of fibrous tissue, preventing bone ingrowth, and decreasing the longevity of secondary fixation.^20^ Therefore, fixation stability is essential for the long‐term performance of press‐fitted implants.

Multiple small press‐fitted pegs have the ability to resist shear forces^21^ and reduce the effects of rocking horse movement because they may be placed near the periphery of the glenoid^22^ so that they anchor in stiffer and denser subchondral bone instead of the deeper trabecular bone near the center.^23^, ^24^, ^25^, ^26^, ^27^ Pull‐out tests have been used to assess fixation stability of bone screws^28^ or cemented pegs^16^ but data on the performance of small press‐fitted UHMWPE pegs is currently unavailable.

This study investigated the effects of bone density, interference fit and peg geometry on the primary fixation of small press‐fitted pegs. The purpose was to obtain data that would be useful for improving the cementless fixation of UHMWPE glenoid components.

## METHODS

**Figure 1:**
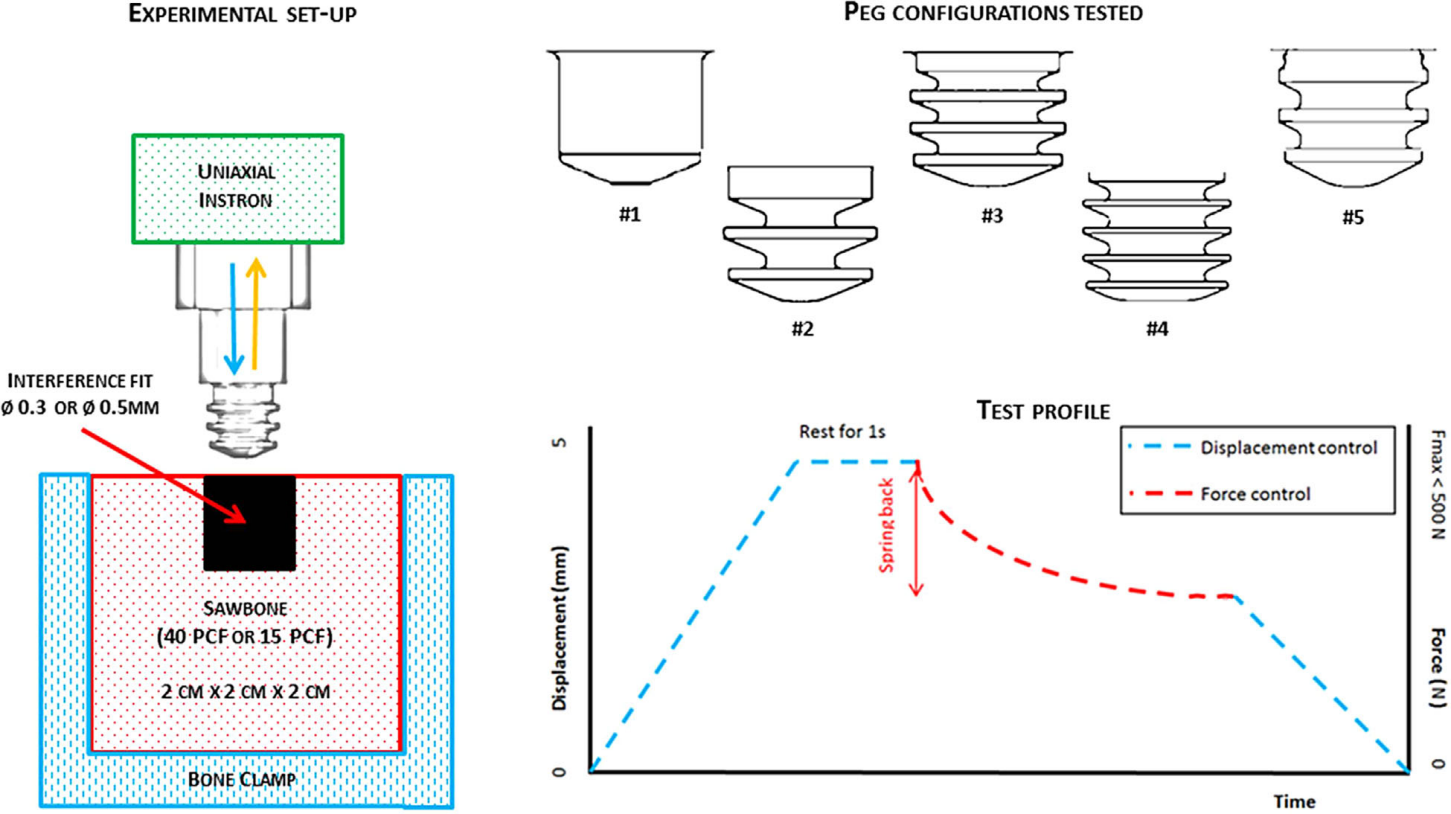
Left: Experimental set‐up for pull‐out tests—a peg specimen is mounted into a uniaxial Instron and pushed into a 2 × 2 × 2 cm^3^ clamped Sawbone block (40 or 15 PCF) with a 0.3 or 0.5 mm diametral interference fit. Top right: The five peg geometries tested. Bottom right: Test profile for the push‐in/pull‐out test which was split into three parts: push‐in of the peg in displacement control, measurement of spring‐back in force control and pull‐out in displacement control.

Five different small press‐fitted peg geometries were manufactured out of the same 7 mm diameter UHMWPE rod (*E* = 750 MPa, Davis Industrial Plastics Ltd, Crawley, United Kingdom). These were 5 mm diameter and length. A computational parametric finite element analysis of peg design performed by the authors^22^ tested over 1,000 different glenoid fixation configurations (ranging from keeled to pegged implants, with large and small fixation features distributed either centrally, peripherally, or both) and concluded that configurations with such pegs distributed peripherally in the shallow subchondral bone of the glenoid could provide the best resistance to edge loading while still allowing for bone preservation. They were either plain cylinder pegs (#1) or with a number of fins ranging between fin 3 and 5 (#2–5) (Fig. 1, top right), varying fin aspect ratios (ratio between width and length) and two core diameters (2.2 mm for geometry #2 and 3.2 mm for geometries #3–5). A fillet radius of 0.2 mm connected the peg and its base.

The pegs were mounted into a single‐axis screw‐driven Instron 5565 material testing machine (Instron, High Wycombe, UK) through a threaded stainless steel fixture (Fig. 1, left). The pegs were pushed into a foam block 5 mm axially under displacement control at 1 mm/s and a maximum force limit set at 500 N. The maximum push‐in (*P*_in_) force was recorded and followed by a 1 s resting period. The test was then switched to force control, the force reduced to zero and spring‐back elastic displacement measured approximately via crosshead movement. The specimen was then pulled out axially for 5 mm at a rate of 1 mm/s and the maximum pull‐out force (*P*_out_) recorded. The test profile is depicted in Figure 1 (bottom right).

A total of 16 specimens for each peg geometry were tested for two types of polyurethane foam blocks (Sawbones, Pacific Research Laboratories, Inc., WA) representing hard (40 PCF, 0.64 g/cm^3^, 759 MPa, Sawbones #1522‐05) and soft (15 PCF, 0.24 g/cm^3^, 123 MPa, Sawbones #1522‐02) bone. These foam blocks were used as surrogates for high and low density bone, respectively, because of their consistent mechanical properties, homogeneity, and isotropy.^29^ Two different diametral interference fits, 0.3 and 0.5 mm, were studied by pre‐drilling holes with 4.7 and 4.5 mm diameter with a benchtop drill press. All the interference fit tests were performed in the same bone surrogate block in order to minimize variation of polyurethane foam quality. Geometry #2 was then tested for different fillet radii at the peg‐base junction (0.5, 1.0, and 1.5 mm) for hard bone (0.3 mm diametral interference), in order to assess the effects on the spring‐back displacement of the peg. A total of 92 samples were tested: four specimens were tested for each of the 23 combinations of parameters. Three criteria were used to compare the performance of different peg geometries and highlight the best possible combination for fixation stability:

1. Peg specimens should be pushed into hard bone without fracturing and below a maximum *P*_in_ force of 380 N. This value was taken as 10% of the intact glenoid bone ultimate strength as measured by Frich et al.^2^ and deemed to be sufficient to allow the simultaneous insertion of multiple peripheral pegs (up to seven) comfortably without fracturing of the glenoid bone during impaction.
2. Spring‐back displacements should be the lowest possible. Large spring‐back displacements can lead to incorrect seating as the implant is not fully supported by the underlying bone, leading to UHMWPE deformation^17^ and fatigue failure,^7^ larger gaps at the interface for bone to bridge^30^ and increased micromotion^14^ with a heightened risk of loosening.
3. The optimal interference fit should be the one that produces the largest *P*_out_ in soft bone, an indicator of press‐fit strength for that peg. *P*_out_/*P*_in_ ratios were also investigated as when *P*_out_/*P*_in_ > 1, P_out_ forces exceed *P*_in_ forces and larger fixation stability is achieved for pegs with similar insertion forces. The pegs were visually inspected for signs of fracturing and plastic deformation after pull‐out.

Statistical analysis was performed using SPSS software (IBM SPSS Statistics 22.0.0, NY). A three‐way analysis of variance (ANOVA) with Bonferroni post hoc correction multiple comparisons was performed to identify significant differences between the levels of the bone surrogate type, interference fit, and peg design independent variables for *P*_in_, *P*_out_, spring‐back and *P*_out_/*P*_in_ ratio dependent variables. A one‐way ANOVA was also performed for each dependant variable to identify significant differences between peg geometries averaged across the other two independent variables. Significance for both analyses was defined as *p *< 0.05. Data are reported as mean value ± standard deviation (SD).

## RESULTS

**Figure 2:**
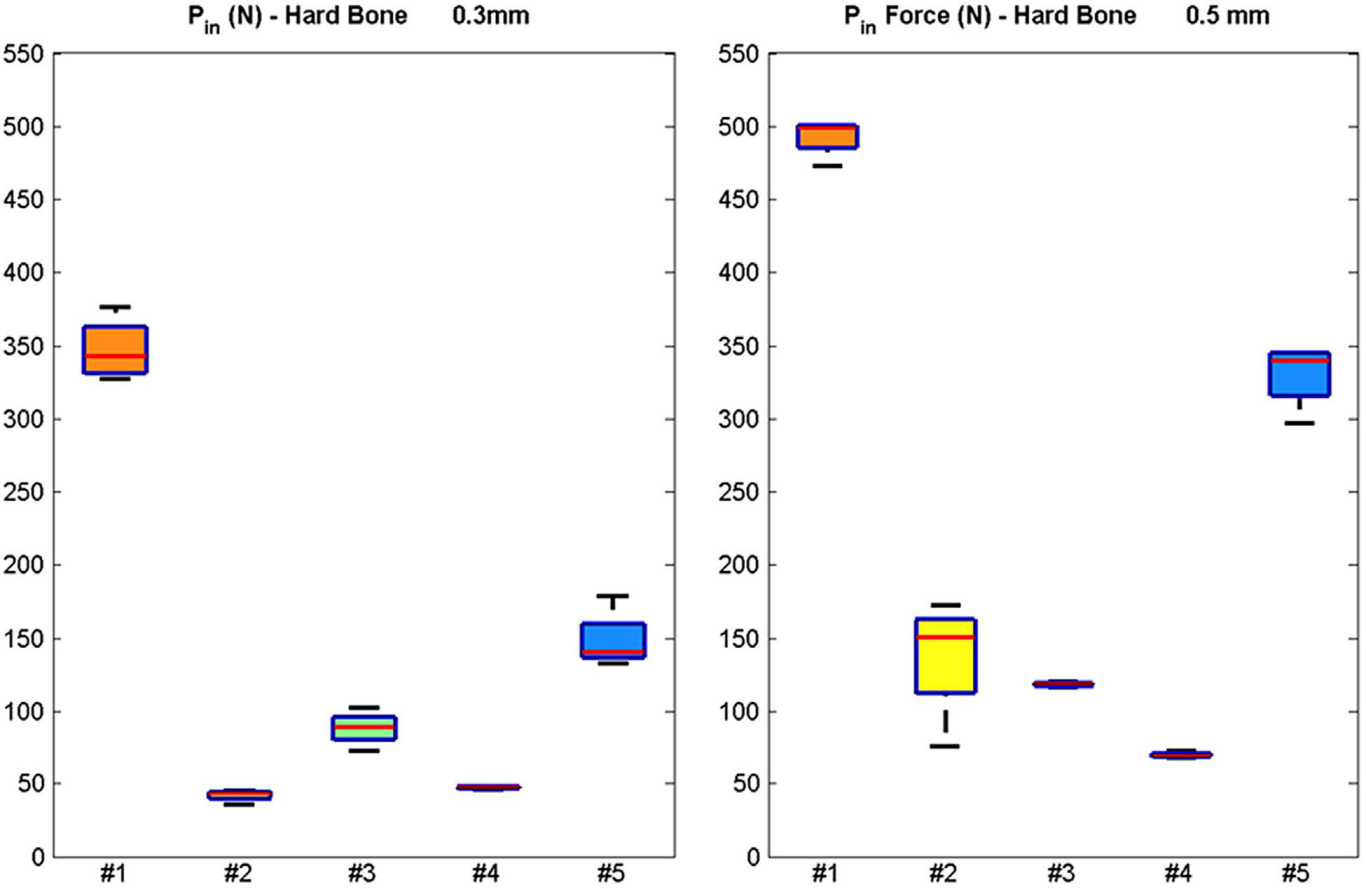
Boxplots of the push‐in force (in N) for the five different geometries tested in hard bone surrogate for interference fits of 0.3 mm (left) and 0.5 mm (right). The red lines indicate the median, the top and bottom box edges correspond to ± 2.7 SD. The black lines extend to the adjacent value, the most extreme data point that is not an outlier.

A boxplot of *P*_in_ forces (in N) for the five geometries tested in hard bone for both interference fits shows that geometry #1 exceeded the limit *P*_in_ force for hard bone (Fig. 2). Significant differences were observed for all designs’ pairwise comparisons (*p* < 0.05) except between geometries #2 and #4. Finned pegs resulted in lower *P*_in_ forces than cylindrical pegs, with the lowest *P*_in_ forces produced by geometries #2, #3, and #4 for both types of bone and interference fits tested. Reducing the interference fit from 0.5 to 0.3 mm was shown to reduce *P*_in_ (*p* < 0.05), with geometries #3 and #4 the least affected by this change. Deformation of the fins was observed after pull‐out for geometries #2, #3, and #4 but no fractures of the UHMWPE were registered.

**Figure 3:**
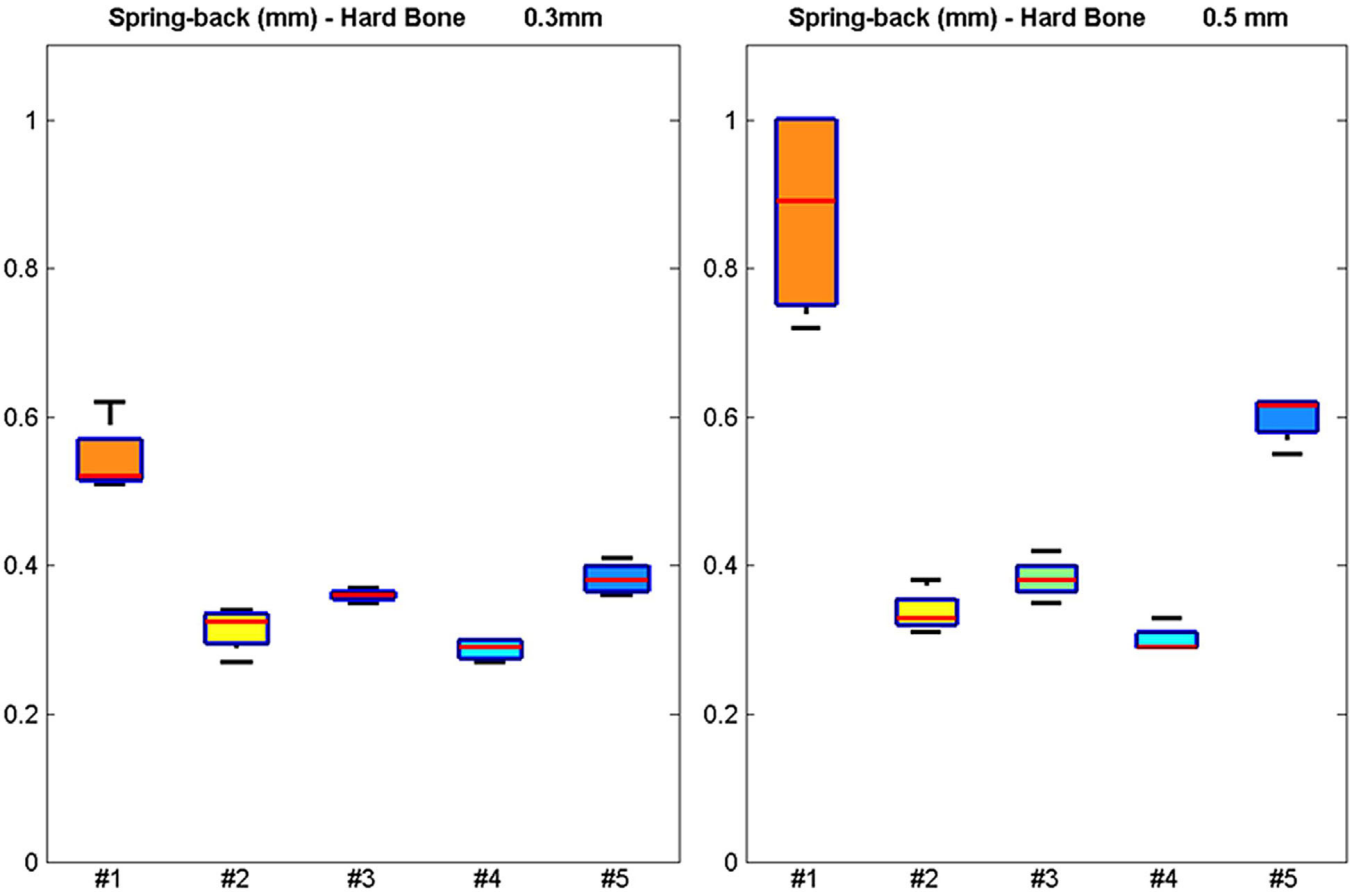
Boxplots of the spring‐back displacement (in mm) for the five different geometries tested in hard bone surrogate for interference fits of 0.3 mm (left) and 0.5 mm (right). The red lines indicate the median, the top, and bottom box edges correspond to ± 2.7 SD. The black lines extend to the adjacent value, the most extreme data point that is not an outlier.

The spring‐back displacements (in mm) for the five geometries tested in hard bone are shown in Figure 3. Geometries #1 and #5 had the largest spring‐back in hard bone with 0.5 mm interference and #1 also doing so for 0.3 mm interference. Bone surrogate type and interference fit were found to influence spring‐back displacements (*p* < 0.05).

**Figure 4:**
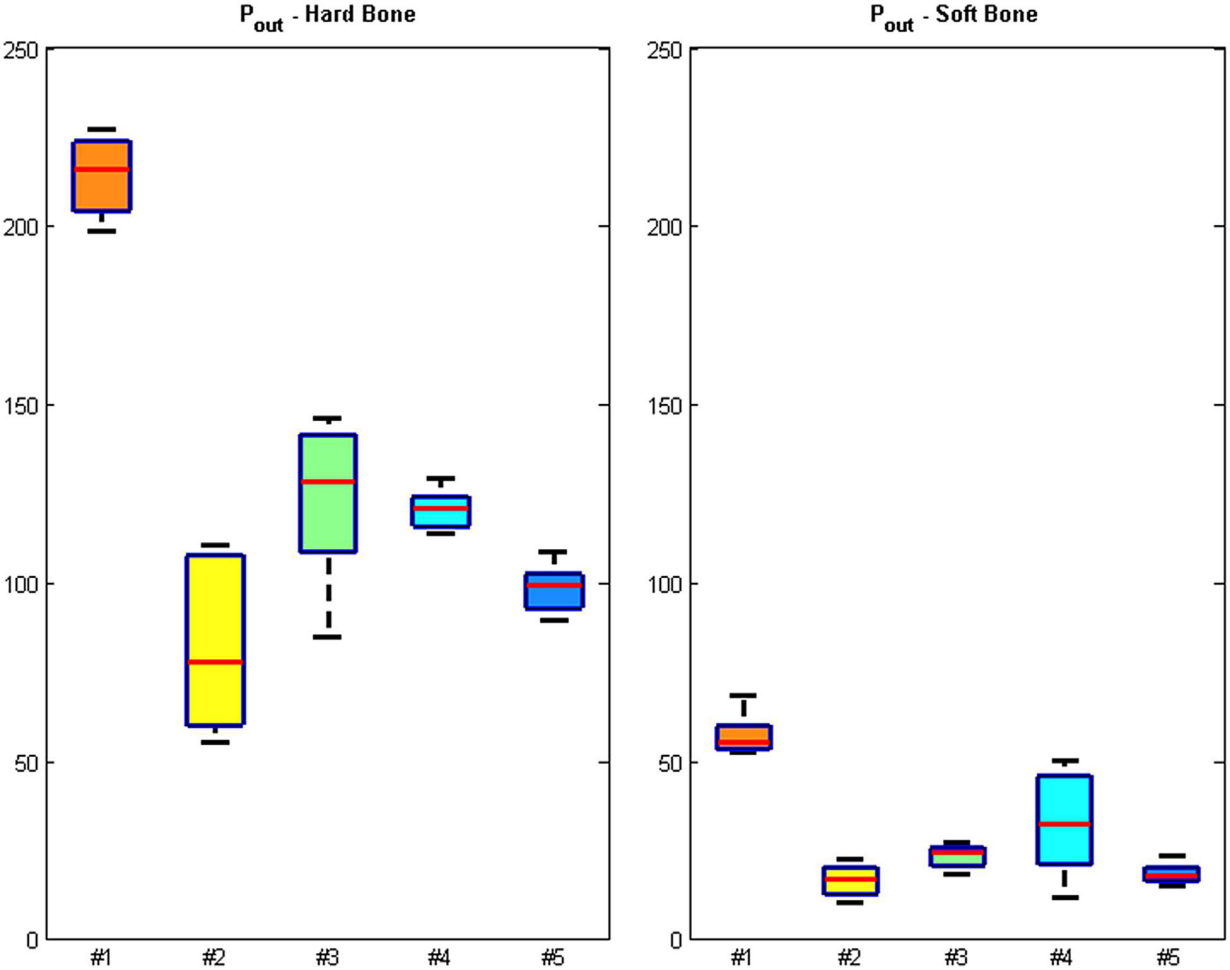
Boxplots of the pull‐out forces ratios (Pout) for the five different geometries tested in hard and soft bone surrogates for interference fits of 0.3 mm (left) and 0.5 mm (right). The red lines indicate the median, the top, and bottom box edges correspond to ± 2.7 SD. The black lines extend to the adjacent value, the most extreme data point that is not an outlier.

Figure 4 shows the *P*_out_ forces for the five geometries for both bone surrogate types. *P*_out_ forces were not significantly affected by the interference fit but depended on bone surrogate density (*p* < 0.05). Geometry #1 produced the largest *P*_out_ forces for both bone surrogates. No statistical significance was found between geometries #2, #3 and #4 for soft bone.

High‐density bone surrogate tests had significantly higher *P*_out_/*P*_in_ than low‐density bone surrogate tests (*p *< 0.05, Table 1). A similar difference was found with increasing interference fit (*p *< 0.05). Geometries #2, #3, and #4 produced larger *P*_out_ forces than *P*_in_ forces (*P*_out_/*P*_in_ > 1) for hard bone surrogates with interference fit of 0.3 mm (1.4, 1.2, and 2.4, respectively). Geometry #4 produced the largest *P*_out_/*P*_in_ ratios and no statistical significance was found between geometry #2 and #3.

**Table 1:** Mean Values (±  Standard Deviation, SD) for Average Push‐In Force (*P*_in_), Pull‐Out Force (*P*_out_), Spring‐Back and Pull‐Out/Push‐In Ratio (*P*_out_/*P*_in_) for the Five Peg Geometries Tested in Soft and Hard Bone Surrogates and Diametral Interference Fit of 0.3 and 0.5 mm

Geometry	Bone Type	Interference Fit	Average *P*_in_ (N)	Average *P*_out_ (N)	Average Spring‐Back (mm)	Average *P*_out_/*P*_in_
#1	Hard	0.5	484.6^a^,^b^	223.9^a^	0.75^a^	0.46^a^
		0.3	346.8 (± 18.7)	209.1 (± 9.4)	0.54 (± 0.04)	0.60 (± 0.00)
	Soft	0.5	73.7 (± 4.4)	60.7 (± 5.4)	0.34 (± 0.00)	0.82 (± 0.05)
		0.3	59.3 (± 4.7)	54.1 (± 1.5)	0.27 (± 0.03)	0.93 (± 0.08)
#2	Hard	0.5	137.6 (± 36.5)	104.6 (± 6.8)	0.34 (± 0.03)	0.82 (± 0.23)
		0.3	42.8 (± 3.5)	59.7 (± 3.0)	0.32 (± 0.03)	1.40 (± 0.07)^c^
	Soft	0.5	43.7 (± 2.5)	20.7 (± 1.5)	0.35 (± 0.05)	0.48 (± 0.05)
		0.3	19.1 (± 0.7)	12.9 (± 1.9)	0.29 (± 0.01)	0.68 (± 0.08)
#3	Hard	0.5	118.6 (± 1.4)	140.2 (± 5.4)	0.38 (± 0.02)	1.18 (± 0.06)^c^
		0.3	88.4 (± 10.5)	106.6 (± 14.4)	0.36 (± 0.01)	1.20 (± 0.04)^c^
	Soft	0.5	58.3 (± 1.1)	26.1 (± 0.9)	0.34 (± 0.02)	0.45 (± 0.01)
		0.3	28.2 (± 2.1)	20.9 (± 1.9)	0.29 (±0.03)	0.74 (± 0.03)
#4	Hard	0.5	70.2 (± 1.6)	122.9 (± 5.8)	0.30 (± 0.02)	1.75 (± 0.08)^c^
		0.3	48.0 (± 0.9)	115.1 (± 8.4)	0.29 (± 0.01)	2.40 (± 0.14)^c^
	Soft	0.5	51.8 (± 3.0)	46.1 (± 2.9)	0.39 (± 0.03)	0.89 (± 0.05)
		0.3	26.8 (± 2.1)	19.2 (± 4.2)	0.18 (± 0.10)	0.71 (± 0.13)
#5	Hard	0.5	330.3 (± 19.7)	100.6 (± 7.0)	0.60 (± 0.03)	0.31 (± 0.04)
		0.3	148.3 (± 18.0)	91.5 (± 12.5)	0.38 (± 0.02)	0.62 (± 0.07)
	Soft	0.5	58.4 (± 2.0)	18.5 (± 1.9)	0.35 (± 0.01)	0.32 (± 0.04)
		0.3	28.4 (± 1.7)	18.9 (± 3.1)	0.28 (± 0.05)	0.66 (± 0.08)

^a^ Average values calculated for two tests only as *P*_in_ exceeded 500 N, therefore SD is not reported.

^b^ Exceeding the limit of 380 N.^1^

^c^ *P*_out_/*P*_in_ larger than limit of 1 when pull‐out forces exceed push‐in forces.

High repeatability among samples of the same geometry tested is noted, with all Pearson's product‐moment correlation coefficients between curves ranging between 0.747 and 0.998 (*p* < 0.0001). The mean values (± SD) for average *P*_in_ force, *P*_out_ force, spring‐back and *P*_out_/*P*_in_ ratios are reported in Table 1. Two tests were stopped because they exceeded the maximum *P*_in_ value of 500 N (geometry #1, hard bone, 0.5 mm interference). Force‐displacement curves for all specimens tested are included in the electronic supplementary material. The influence of fillet size was tested for geometry #2 and the results for 0.5, 1.0, and 1.5 mm fillet radii are presented in Table 2. Increasing radius of fillet led to a linear increase of spring‐back displacements (*r* ^2^ = 0.546, *p *< 0.05), reducing *P*_in_ and *P*_out_ forces but without significant change to the *P*_out_/*P*_in_ ratio.

**Table 2:** Mean (±  Standard Deviation, SD) Values for Average Push‐In Force (*P*_in_), Pull‐Out force (*P*_out_), Spring‐Back and Pull‐Out/Push‐In Ratio (*P*_out_/*P*_in_) for Geometry 2 With Three Different Fillet Radii: 0.5, 1.0, and 1.5 mm

Fillet Type	*P*_in_ (N)	*P*_out_ (N)	*P*_out_/*P*_in_	Spring‐back (mm)
0.5 mm	68.9 (± 9.1)	93.4 (± 16.9)	1.35 (± 0.14)	0.68 (± 0.03)
1.0 mm	65.4 (± 10.5)	88.5 (± 9.1)	1.39 (± 0.28)	0.72 (± 0.04)
1.5 mm	61.2 (± 6.6)	81.1 (± 14.0)	1.32 (± 0.18)	0.77 (± 0.05)

## DISCUSSION

The most important finding of this study was that geometries with lower stiffness fins (or large fin length to width aspect ratio) were the best performing designs in terms of primary fixation stability for small press‐fitted UHMWPE pegs. They require the lowest force to fully seat, meaning they are less damaging to the bone during implantation. These pegs provide the highest *P*_out_/*P*_in_ ratio, indicating that when implanted they provide the strongest anchoring for the glenoid component. These findings are relevant for the design of cementless implants because primary fixation stability has been shown to be an important factor in achieving osseointegration and longevity of secondary fixation^20^ and glenoid components with direct contact between the UHMWPE and bone are commercially available.^31^

Lower *P*_in_ forces were observed in the pegged components where deformation of the fins was observed (#2, #3 and #4) for both densities of bone surrogate and interference fits tested (Table 1). This indicates that fin stiffness is an important criterion in the prevention of possible bone fractures and needs to be taken into consideration, particularly when designing components relying on multiple pegs. It was not possible to define whether the deformation observed was produced during insertion or extraction. However, this is still a useful observation as it provides evidence of the resistance of peg geometries during implantation life. This is further highlighted when the *P*_in_ forces of geometry #2 and #5 are compared for hard bone (Fig. 2). Despite having the same number of fins, the forces produced were much lower for the fins of geometry #2, which had a larger radial distance from the core and, therefore, lower resistance to bending. However, reducing the central core diameter reduces the peg's cross sectional area. This can reduce resistance to shear, which may result in peri‐prosthetic fracture after traumatic events.^1^, ^8^ These are competing requirements that need to be balanced in implant design.

Figure 4 highlights that *P*_out_ forces were not significantly affected by the interference fit but dependent on bone surrogate type (*p* < 0.05). The cylinder‐shaped peg geometry #1 produced the largest *P*_out_ forces for both bone surrogates because of the high contact area with bone surrogate. No statistical significance was found between geometries #2 and #3. *P*_out_ forces were lower than what has been observed in cemented pegs.^16^ This is to be expected as cemented fixation is by nature at its strongest immediately after setting of the cement, whereas cementless fixation is at its weakest immediately after implantation when osseointegration has not started. This further highlights the need for a stable primary fixation in cementless shoulder arthoplasty.

Bone surrogate density and interference fit were observed to influence spring‐back displacements significantly (*p* < 0.05). Geometries #1 and #5 produced considerably larger spring‐back displacements in the tests in hard bone with 0.5 mm interference, while #1 also doing so with 0.3 mm interference. No significant differences were found between geometries #2, #3, and #4. This implied that the stiffness of the fixation features can affect implant seating, because fins with large length to width aspect ratio have lower yield strength. This promotes plastic deformation of the fins which in turn allows the peg to be pushed with a lower elastic spring‐back response as they remain tightly flexed against the fixation hole. This has also been verified in other biomechanical studies.^32^ We have shown experimentally that such gap can significantly increase interface micromotion beyond what is commonly accepted as a threshold of osteointegration.^14^ It was also observed that increasing the radius of the fillet between the peg and the implant base can lead to an increase of spring‐back displacements, as contact between the fillet and the bone prevented the peg from being pushed in further (Table 2). *P*_in_ and *P*_out_ forces decreased with increasing fillet radius as the peg is in contact with less foam area, but the *P*_out_/*P*_in_ ratio remained unchanged. Drilling of chamfered holes could minimize spring‐back displacements. This finding is of clinical importance, as it can contribute to the improvement of surgical instrumentation for preparation of the fixation surface.

Hard bone surrogate resulted in increased *P*_out_/*P*_in_ ratios in comparison to soft bone surrogate (*p* < 0.05) (Table 1). A similar statistical significance was found with changes in interference fit (*p* < 0.05). Geometries with plastic deformation of the fins produced larger *P*_out_ forces than *P*_in_ forces for hard bone blocks. A reduction of up to 70% of *P*_out_/*P*_in_ ratio was found by reducing the interference fit. This is due to the lower *P*_in_ forces produced as the hole clearance increases and friction between the peg and the bone surrogate is reduced (Table 1). This implies that unintended changes to the planned interference fit during bone preparation in surgery could have a big impact in the fixation stability of UHMWPE components.

Several limitations to this study need to be considered. A total of 92 samples were tested: 80 samples for all possible 20 combinations of bone surrogate quality (soft and hard), diametral interference fit (0.3 and 0.5 mm) and peg design (five geometries) and a further 12 samples for the three different fillet radii. A sample size of *n* = 4 was selected for each combination because simplicity and speed of testing were important in comparing across this broad range of parameters. This sample size is in agreement with similar work for cemented pegs in natural glenoid bones^16^ and high repeatability among samples was observed as a result of strict constraints being placed on all testing parameters. Nevertheless, this study should be considered as a screening assessment to highlight which best performing geometries should be tested further in more detail and larger numbers. Having established the peg geometry that best performs in terms of fixation stability, further work will focus in building patient‐specific finite element models in order to investigate in more detail their potential in reducing rocking horse movement and improving osseointegration. These will be coupled with cadaveric studies for validation and exploration of such implants’ performance in physiological conditions using methodology previously established.^14^

Solid rigid polyurethane foam blocks were used as bone surrogates and chosen as their uniformity allows for simultaneous comparison of different parameters. We believe it to be an appropriate substitute as its material properties are similar to what has been measured for the glenoid bone (67–400 MPa and 0.14–0.48 g/cm^3^)^23^, ^26^, ^33^ and such surrogates have previously been used in other fixation stability studies.^28^, ^29^

Finally, this study analyzed the fixation stability of different peg geometries under tensile axial loading. The pull‐out rate tested was six times faster than a similar study on cemented pegs^16^ and deemed to be an appropriate worst case scenario. The use of a slower pull‐out rate could have changed the plastic deformation behavior of the pegs but given that the dynamic coefficient of friction is independent of the sliding speed, it would not have resulted in different interface friction between the pegs and the polyurethane foam. Continuous tensile forces pulling out the press‐fitted pegs are unlikely to occur in vivo due to constant alterations of force magnitude and direction as the humeral component translates on the glenoid surface.^34^ This complex loading of the shoulder fixation is not considered in this study, but *P*_out_ forces magnitudes and *P*_out_/*P*_in_ ratios can still be taken as an indicator of the fixation stability of peg geometries^16^ as their relationship with peg geometry, fin aspect ratio, and resistance to pull‐out forces is shown in this study.

In conclusion, press‐fitted peg geometries with low stiffness fins were the best performing in terms of fixation stability. Fin stiffness is highlighted as an important factor in primary fixation stability and the prevention of possible bone fractures. Plastic deformation of the fins allows for a lower elastic spring‐back response, reducing incorrect seating of the glenoid implant and subsequent loosening. The decision on the degree of importance to attribute to each fixation criterion should be specific to the intended application of the design in question. This combination where push‐in force and spring‐back displacement is minimized and the ratio between the pull‐out and push‐in forces is maximized was chosen as it provides a stable primary fixation. This ensures damage to bone is kept to a minimum during implantation while providing the implant with resistance to increase in interface micromotion resulting from edge loading that can compromise secondary fixation and longevity of the cementless fixation. The use of countersinking collars is proposed to minimize the effects of incorrect bone preparation on the primary fixation of press‐fitted pegs and unintended changes to the planned interference fit during bone preparation in surgery was shown to impact the fixation stability of UHMWPE components.

## AUTHORS' CONTRIBUTIONS

We confirm that these results have not been published elsewhere and are not under consideration by any other journal. All authors have read and approved the manuscript and agree with its submission to the Journal of Orthopaedic Research. Specific contributions: Research design (DMG, UH, JJ, AAA), methods development (DMG), data analysis (DMG), data interpretation (DMG), study supervision (UH, JJ, AAA), manuscript preparation (DMG), manuscript editing (DMG, UH, JJ, AAA), manuscript approval (DMG, UH, JJ, AAA).

## Supporting information

Additional supporting information may be found in the online version of this article.

Supporting Figure S1.
