# Supplementary figures and images for: Stability of small pegs for cementless implant fixation
Source: J Orthop Res. 2017 May 23;35(12):2765–72. doi: 10.1002/jor.23572 (PMC5763372; doi:10.1002/jor.23572)

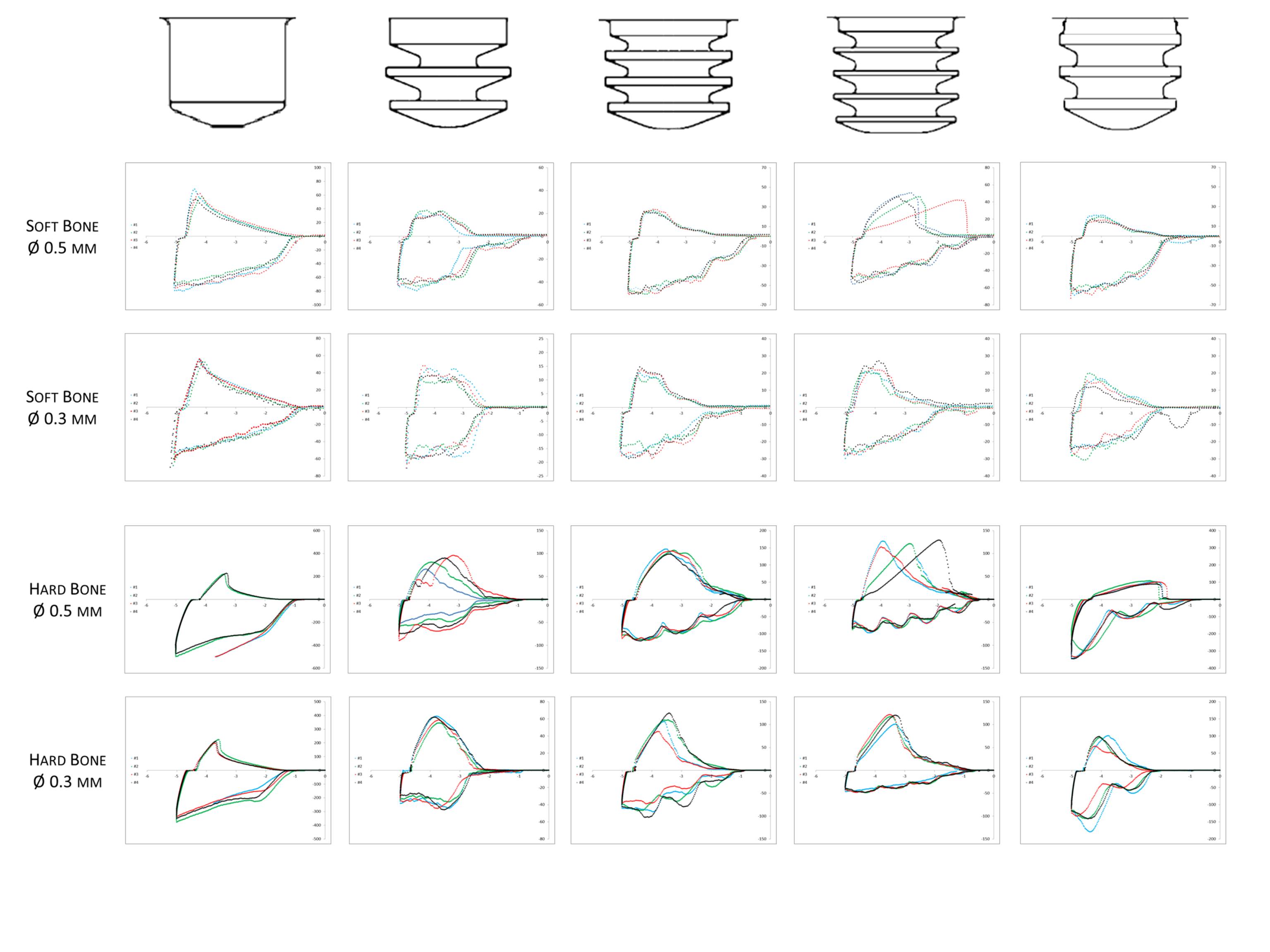

Supplement: Supplementary file 1 — Supporting Figure S1. [file JOR-35-2765-s001.JPG]
